# Exploring methods for generating synthetic data in Scotland to improve access to public sector data for research

**DOI:** 10.23889/ijpds.v9i5.2738

**Published:** 2024-09-18

**Authors:** Aileen Grieve, Nora Cooke O'Dowd

**Affiliations:** 1 Research Data Scotland

## Objective and Approach

We are working to improve the economic, social and environmental wellbeing in Scotland by enabling access to and linkage of public-sector data for research in the public good. Our objective was to examine how synthetic data can support this aim by allowing researchers to test approaches and write code while awaiting the necessary permissions to use the real data. We have explored generating synthetic data from metadata and from actual data.

## Results

Through engagement, researchers expressed interest in low fidelity data, which minimises privacy and information governance risks. We have created low fidelity data from information provided by data controllers in publicly-available data dictionaries. We have also created more high-fidelity data based on actual data, using the synthpop tool. We are carrying out further work to engage with data controllers about the level of fidelity that is acceptable to them, as well as the information that needs to be included in the data dictionary to meet this in an automated way, and how we will share datasets with researchers. A key part of these considerations is the ability to link synthetic datasets and the impact that has on the fidelity of the data.

## Conclusions and Implications

We will share our approaches, the limitations we have found in using metadata to create synthetic data, difficulties encountered in using the synthpop tool, engagement we have had from controllers and researchers, and the proposed way forward for synthetic data in Scotland.

